# Human Papillomavirus Vaccine immune responses in an Olive baboon model is not compromised by chronic *Schistosoma mansoni* infections

**DOI:** 10.3934/microbiol.2025030

**Published:** 2025-08-07

**Authors:** Linda Obiero, Edinah Songoro, Martin Omondi, Ruth Nyakundi, Lucy Ochola

**Affiliations:** 1 Jomo Kenyatta University of Agriculture and Technology; 2 Kenya Institute of Primate Research

**Keywords:** Human papillomavirus, HPV vaccine, chronic *schistosomiasis*, innate immune cells, immune cells antibodies

## Abstract

Human papillomavirus (HPV) vaccines elicit specific serum antibodies that confer long-lasting protection and may reduce HPV-related cancers. However, helminthic infections such as chronic schistosomiasis infection at the time of HPV vaccination are known to alter immune responses. This study investigated the impact of chronic *S. mansoni* infection on immune responses to the HPV vaccine in olive baboons. Baboons were assigned to three groups: (1) untreated *S. mansoni*-infected, (2) *S. mansoni*-infected and treated with Praziquantel, and (3) uninfected controls. All received two doses of the Cervarix HPV vaccine four weeks apart. Immune responses were measured using flow cytometry and ELISA. Gardasil® quadrivalent HPV vaccine antigen was used in both ELISA and PBMC stimulation for cytokine ELISA supernatants. HPV-specific whole IgG levels significantly increased in all groups except for the untreated *S. mansoni*-infected group, whose increase was significant after the second dose. Similarly, IgG1 levels increased only after the second dose. There was no significant difference between the treated and untreated infected groups. These findings emphasize the importance of a booster dose for strong antibody production and suggest that a delayed HPV-specific whole IgG response in untreated *S. mansoni*-infected individuals reinforces the need for booster HPV vaccination in endemic regions. The results affirm the vaccine's effectiveness in *S. mansoni* endemic areas.

## 1. Introduction

Persistent HPV infections are known to cause up to 95% of cervical cancers, the fourth most common cancer type affecting women globally, responsible for 350,000 deaths in 2022. Nearly 70% of cervical cancer cases are attributed to HPV types 16 and 18 [1]. There are currently six approved HPV vaccines: one nanovalent, two quadrivalent, and three bivalent vaccines. All six vaccines are highly effective in preventing infections with HPV types 16 and 18. These include Cervarix, a bivalent HPV vaccine, and Gardasil, a quadrivalent HPV vaccine. The bivalent *Cervarix* vaccine contains antigen concentrations of HPV18 and HPV16, which protects against HPV 18 and HPV 16 [2]. The Quadrivalent *Gardasil* vaccine (Gardasil, Merck & Co.) was introduced at the launch of the Kenyan HPV vaccine initiative in 2019 and confers protection against HPV 18, 16, 11, and 6 [3],[4]. These HPV vaccines utilize highly immunogenic virus-like particles (VLPs) containing the L1 capsid protein marked by the production of type-specific neutralizing antibodies, which suggests high efficacy against HPV infections [5].

HPV vaccines elicit protective immunity by stimulating innate and adaptive antiviral defense mechanisms. and natural killer cells aid the antigen presentation, essential for the innate immune responses [6], and the production of interleukin-1 (IL-1) and tumor necrosis factor-alpha (TNF-α) pro-inflammatory cytokines [7]. T helper cells aid B-cell activation and subsequent high levels of antibody production [8], while CD8^+^ T cells function in direct viral clearance [9]. Anamnestic HPV responses are established by the persistence of memory cells, highlighting the importance of innate and adaptive cell-mediated immunity, which is crucial for the protection against HPV infection and HPV vaccine immune responses. In healthy humans, total monocytes are 10–20% of PBMCs [10] with monocyte subset frequencies as 85% for classical monocytes, 5% for intermediate monocytes, and 10% for non-classical monocytes [11],[12]. Natural killer cells make up 5–20% of lymphocytes, while CD4+ and CD8+ are at a ratio of 1:2 of CD3+ lymphocytes [10]. In baboons, standard references for the enumeration of PBMCs is yet to be documented. However, the homology in immune function of non-human primates (NHPs) and humans, enables the use of NHPs as animal models for immune response studies [6],[7].The outcome of HPV vaccination is the production of neutralizing IgG antibodies [13]. However, immune correlates for HPV vaccination are yet to be identified [14],[15].

Several studies, including a single-dose efficacy study in Kenya and Tanzania, confirm the vaccine's long-lasting immunity [8]–[13]. HPV vaccines confer protection against HPV infections and show no evidence of decreased long-lasting protection over time [14]. However, helminthic infections such as schistosomiasis are known to impair these immune responses [15].

Schistosomiasis, a parasitic disease caused by *Schistosoma* species, is prevalent worldwide, with over 240 million cases reported by the World Health Organization in 2024 [16], with Africa bearing the greatest burden of 85% of schistosomiasis incidences [17]. Schistosomiasis infection is known to initiate a complex host immune response involving innate and adaptive immunity [18].

Acute schistosomiasis triggers Th1 responses characterized by tumor necrosis factor (TNF)-α, interleukin-1β (IL-1β), IL-6, IL-12, tumor necrosis factor (TNF)-α, and interferon (IFN)-γ, which support macrophage activation and cell-mediated immunity[19]. The chronic phase of schistosomiasis infection induces a Th2 response characterized by IL-4 that aids in controlling infection but can contribute to fibrosis and granuloma formation, modulated by T regulatory (Tregs) cells and IL-10 [19],[20]. The resulting immunomodulation of chronic *Schistosoma* infections can alter vaccine-induced immune responses, potentially reducing the efficacy of vaccines such as those for the human papillomavirus (HPV), an etiological virus for cervical cancer [21].

Several human studies have shown that *S. mansoni* impairs vaccine immune responses for the tetanus toxoid vaccine, BCG vaccine, candidate TB vaccine, polio vaccine, and HPV vaccine [15],[22]–[25]. In a nonhuman primate study, an ongoing, chronic *S. mansoni* infection at the time of vaccination resulted in a reduction of HPV-specific IgG antibodies, suggesting a compromised protective effect of the vaccine. However, research has shown that early anti-helminth treatment can reverse the immunomodulation caused by parasite antigens in non-human primates [11] and in human subjects [15].

Studies evaluating HPV vaccine immunogenicity in helminth-exposed populations have shown varied results [15],[26]. The effects of chronic schistosomiasis on innate immune and adaptive HPV-specific immune responses are yet to be adequately investigated. This study was based on a previous bivalent HPV vaccine immune response study [11], which investigated whole IgG, IL-4, IFN-γ, and lymphoproliferation. Therefore, this study was designed to evaluate broader HPV vaccine-specific innate and adaptive immune responses in chronic and treated *S. mansoni* infections using a baboon model, a natural host of *S. mansoni*.

## 2. Materials and methods

### 2.1. Study design

This study is mounted on a previous HPV vaccine immune response study [11], and utilizes archived samples. Previously, ten olive baboons were randomly placed into three groups: Group 1: Schisto + HPV vaccine (n = 3), Group 2: Schisto + PZQ + HPV vaccine (n = 4), and Group 3: HPV vaccine only (n = 3).

Group 1 and Group 2 animals were infected with 500 *S. mansoni* cercariae, and the infection was allowed to the chronic phase. At the onset of chronic infection, at weeks 13 and 14, Group 2 was treated with Praziquantel (PZQ), at a dosage of 80 mg/kg (PZQ; Balcitricide, Bayer Schering Pharma). At week 64 post *schistosomiasis* infection, all three animal groups received two doses of 0.5 mL of two (Cervarix™, GlaxoSmithKline) four weeks apart (Figure S1). Samples collected in this study were archived at −20 °C for serum samples, and peripheral blood mononuclear cells (PBMCs) were cryopreserved at −196 °C in liquid nitrogen.

**Figure 1.**
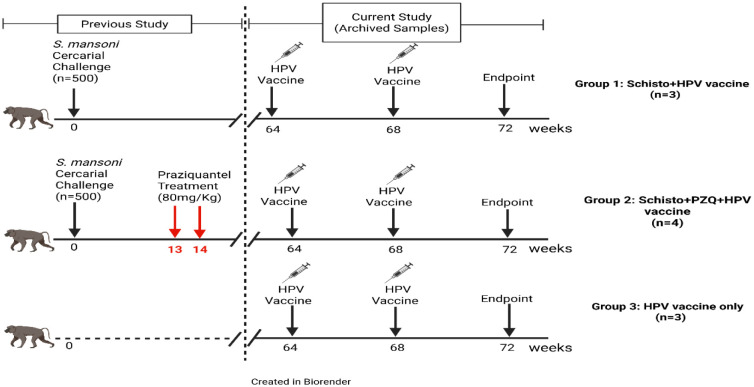
Study design. Archived samples from three animal groups were used in this study: Group 1 animals were chronically infected with *S. mansoni* followed by administration of the Cervarix HPV vaccine (Schisto + HPV vaccine; n = 3). Group 2 animals were initially infected with *schistosomiasis* and were treated with PZQ, followed by vaccination (Schisto + PZQ + HPV vaccine; n = 4). Group 3 animals received the HPV vaccine only (HPV vaccine only; n = 3). Archived serum samples used in this study were collected at T_0,_ before the administration of the 1st dose of the HPV vaccine; at T_1_, before the administration of the 2nd dose of the HPV vaccine; and at Endpoint, four weeks after the second HPV vaccine dose was administered (T_2_). PBMC samples used in the study were collected four weeks after the second HPV vaccine dose was administered (T_2_).

In this current study, archived serum samples used were collected at three study time points: i) at T_0_, before the first HPV vaccine dose was administered; ii) at time point one (T_1_), four weeks after the 1st dose of HPV vaccine was administered; iii) and at T_2_, four weeks after the 2nd HPV vaccine dose was administered (see Figure 1). These serum samples were measured for the whole IgG and IgG subtype responses. To measure specific HPV cellular immune responses, Peripheral Blood Mononuclear Cells (PBMCs) collected four weeks after the second HPV vaccine dose was administered (T_2_) were used.

### 2.2. Measurement of immune responses

#### 2.2.1. Antibody assay

Serum samples were analyzed for HPV-specific whole IgG and IgG subclass antibodies (The Binding Site, Birmingham, UK) using an Enzyme-Linked Immunosorbent Assay (ELISA). Ninety-six-well ELISA plates (Corning®) were coated in duplicates with 50 µL per well of Gardasil-4 HPV vaccine antigen (Gardasil®, Merck & Co.), at a concentration of 1.25 µg/mL for IgG, 2.5 µg/mL for the IgG subclasses. The plates were incubated overnight at 4 °C and washed five times in 0.05% Tween-20 in 1× PBS (PBST) wash buffer. Blocking buffer containing 3% Bovine Serum Albumin (U-CyTech biosciences, Netherlands) in PBS-T (BSA in PBS-T) was added to each well at 100 µL and incubated for 1 hour at 37 °C. Into each well, 50 µL of diluted samples (dilution buffer, 0.5% BSA, PBS-T) were added at ratios of 1:200 for IgG and 1:100 for IgG1. Negative wells (dilution buffer) and blank wells were included. The plates were incubated at 4 °C overnight followed by washing five times in wash buffer, and then 50 µL of secondary antibody was added to each well. The secondary antibody dilutions were 1:2000 for IgG and 1:1000 for IgG subclasses. The plates were incubated for 1 hour at 37 °C. The plates were washed 5 times in wash buffer, and 50 µL of the substrate TMB (3,3,5,5-tetramethylbenzidine, (U-CyTech biosciences, Netherlands) was added to each well, and the color was allowed to develop for 20 minutes with final addition of stop solution (U-CyTech biosciences, Netherlands) was added. The plates were read using the ELISA reader (Biotek Elx808) at 450 nm. Antibody levels were measured as mean optical densities. The mean of the blanks (background) was subtracted from the response optical densities.

#### 2.2.2. PBMC retrieval and culture

Archived PBMC samples were retrieved from liquid nitrogen, quickly thawed at 37 °C, and washed twice in 5 mL of RPMI 1640. The cells were resuspended in 2 mL of complete media (RPMI-1640; Sigma-Aldrich, St. Louis, MO, 1% HEPES; Sigma-Aldrich, Dorset, UK, 10% FBS; Gibco, Canada, 1% L-glutamine; Fisher Biotech, Wembley, Australia, 1% Gentamycin; Sigma-Aldrich, Dorset, UK) and DNase I at 100 µg/mL to prevent PBMC aggregate formation. The cells were incubated at 37 °C for 1 hr., washed to eliminate DNase I, and re-suspended in 2 mL complete media. The cells were further acclimatized for one hour at 37 °C, 5% CO_2_. Following acclimatization, the cells were washed in 1x PBS, and viability was determined using Trypan Blue dye exclusion staining (Sigma-Aldrich, USA T8154, Lot no. RNBB3340). The cells were resuspended at a concentration of 1 × 106 cells/100 µL and cultured at 37 °C, 5% CO_2_ for 72 hours. The culture conditions included: i) media (med) only for negative control, ii) 1.25 µg/mL HPV antigen (Gardasil®, Merck & Co.). Following the 72-hour culture, supernatants were harvested and stored at −80 °C for cytokine analysis, and cells were recounted and measured for immune profiling.

#### 2.2.3. Immunoprofiling

To determine immune cell profiles, 250,000 cells were suspended in 100 uL of 1× PBS and were stained for flow cytometry. Two panels were designed for the phenotyping of: Natural Killer cells (NK cells) and monocytes with allophycocyanin-CY7 (APC-CY7)-CD3, peridinin chlorophyll protein (PerCP Cy5.5)-CD14, phycoerythrin (PE)-CD16 and fluorescein isothiocyanate (FITC)-CD56; CD4+ and CD8+ T cells with APC-Cy7-CD3, (FITC)-CD4, and (PE)-CD8, (Becton and Dickinson [BD] Biosciences, San Jose, CA). The PBMCs were stained and incubated for 25 min, washed, and re-suspended in 500 mL of 1 x PBS. The cells were read on a Becton Dickinson FACS Calibur Flow Cytometer using FACSComp 5.2.1 software. The cells were analyzed using FlowJo v10.10.0 software (BD, Ashland, OR). The gating strategy for innate and adaptive immune cell responses are provided in supplementary Figure S2.

#### 2.2.4. Cytokine assay

The cell culture supernatants were thawed for cytokine ELISA to determine IL-1β and TNF-α concentrations from PBMCs stimulated with HPV vaccine and unstimulated cells. The assay was carried out according to the manufacturer's instructions (Monkey IL-1β ELISA and Monkey TNF-α ELISA; U-CyTech biosciences, Netherlands), with slight modifications (Gent et al., 2019). Two 96-well ELISA plates (Corning®) were coated with 50 µL of 1:200 coating antibodies and incubated overnight at 4 °C. The plates were washed four times in 1 × PBS, 0.05% Tween-20 (PBST) wash buffer. The wells were blocked with 100 µL of 1% BSA-PBST and incubated for 1 hour at 37 °C. Cell culture supernatants and standards were added at a volume of 50 µL to respective wells. A two-fold serial dilution of the standard solution, ranging from 2000 pg/mL to 31.25 pg/mL, was added to the respective wells. Plates were incubated at 4 °C overnight. After washing four times in wash buffer, 50 µL of a 1:100 dilution of biotinylated detection antibody solution was added to each well and incubated for 1 hour at 37 °C. The plates were washed four times in wash buffer, and 50 µL of a 1:100 dilution of Streptavidin-Peroxidase (SPP) conjugate was added to the wells and incubated for 1 hour at 37 °C. After washing four times in the wash buffer, 50 µL of TMB substrate solution was added to each well and incubated in the dark at room temperature. After 20 minutes of incubation, 50 µL/well of the stop solution was added. The plates were read using a microplate reader (ELx808; Biotex) at 450 nm. The mean of the blanks (background) was subtracted from the response optical densities. Mean cytokine levels were expressed as optical density (OD), extrapolated using the cytokine standard curve, and recorded as net cytokine concentrations (mean of duplicate samples minus the mean of negative controls).

### 2.3. Statistical analysis

All data collected from the study was analyzed using GraphPad Prism software Version 8.00 for Windows (GraphPad Software, La Jolla, California, USA, www.graphpad.com). The Shapiro-Wilk test was used to determine normality, and the Kruskall-Wallis with Dunn's *post hoc* tests were used to analyze all the data. The statistical level of significance was set at P < 0.05.

### 2.4. Ethical statement

This study was reviewed and approved by the JKUAT Institutional Science and Ethical Review Committee (Application approval No. JKU/ISERC/02316/0728).

## 3. Results

### 3.1. Enhanced HPV-specific antibody levels in chronic *schistosomiasis* infection after a second HPV vaccine dose

**Figure 2.**
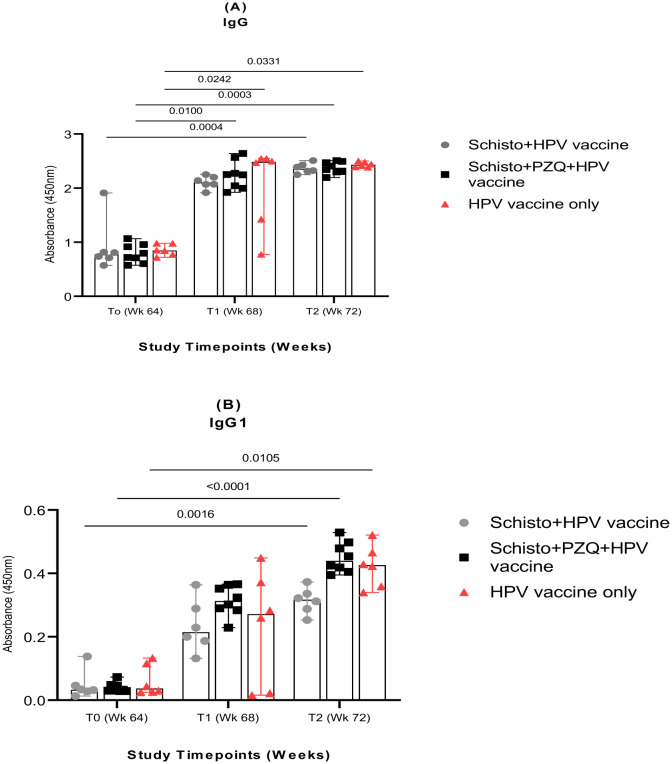
Enhanced HPV-specific antibody levels in chronic *schistosomiasis* infection after a booster HPV vaccine dose. The graph represents HPV-specific whole IgG (A) and IgG1 (B) antibody responses for the Schisto + HPV vaccine, Schisto + PZQ + HPV vaccine, and HPV vaccine-only animal groups. The bars represent each group's mean antibody optical densities at each time point. (T_0_) before administration of the 1st dose of the HPV vaccine, at (T_1_), before administration of the 2nd dose of the HPV vaccine, and at four weeks after the second HPV vaccine dose was administered (T_2_), four weeks after the 2nd HPV vaccine dose was administered. Antibody levels are presented as optical density (OD) measured at a wavelength of 450 nm. Kruskal-Wallis with Dunn's multiple comparison test was done for *post hoc* analysis for all three study groups. Selection criteria for statistical analysis is provided in supplementary Table S1. Descriptive statistics and individual animal data on antibody optical densities are available in Table S2 and Table S3, respectively. Statistical significance was set at P > 0.05 and assigned.

To determine the effect of chronic schistosomiasis on the levels of HPV-specific antibodies, an ELISA assay was undertaken on serum from the three animal groups: Schisto + HPV vaccine, Schisto + PZQ + HPV vaccine, and the HPV-vaccine only group. There was a significant increase in HPV-specific whole IgG levels (Figure 2A) across all time points. We observed two-fold increase in HPV-specific serum whole IgG levels between T_0_ (before vaccination) and T_2_ (4 weeks after the second HPV vaccine dose was administered), across all three animal groups, which was significant as follows: Schisto + HPV vaccine (95% CI: P = 0.0004), Schisto + PZQ + HPV vaccine (95% CI: P = 0.0003), and HPV vaccine only (95% CI: P = 0.0331). Similarly, there was a two-fold increase in HPV-specific serum whole IgG levels between T_0_ (before vaccination) and T_1_ (4 weeks after the first HPV vaccine dose was administered), which was significant as follows: Schisto + PZQ + HPV vaccine (95% CI: P = 0.01) and the HPV vaccine only (95% CI: P = 0.0242). IgG levels did not significantly change between T_0_ (before vaccination) and T1 (4 weeks after the first vaccine dose) in the Schisto + HPV vaccine group, and between the first vaccine dose (T_1_) and the second vaccine dose (T_2_) across all three animal groups. This significant increase observed in HPV-specific whole IgG antibody levels in the Schisto + PZQ + HPV vaccine group compared to the Schisto+HPV vaccine four weeks after the HPV vaccine dose was administered highlights the importance of treating helminth infections before HPV vaccination.

A steady increase in HPV-specific IgG1 (Figure 2B) responses was exhibited from T_0_ to T_1_ of 4.8, 7.9, and 3.8-fold change in the Schisto + HPV vaccine, Schisto + PZQ + HPV vaccine, and HPV vaccine only animal groups, respectively, which was not significant. Similarly, an increase in HPV-specific serum IgG levels between T_1_ and T_2_ was observed as a 1.3, 1.4, and 1.8-fold change for Schisto + HPV vaccine, Schisto + PZQ + HPV vaccine, and HPV vaccine-only animal groups, respectively, which was equally not significant. A delayed HPV-specific IgG1 antibody response after the first HPV vaccine dose was exhibited, and the increase in antibody levels was only significant after the second dose, across all three animal groups. This indicates that a booster dose is essential in HPV-specific IgG1 antibody responses. We observed no significant differences in HPV-specific whole IgG and IgG1 antibody levels between the treated and untreated groups post-vaccination. This was likely impacted by the low sample size of n = 3; n = 4 in the animal groups. IgG2, IgG3, and IgG4 levels remained unchanged across all assessed time points, with optical densities consistently comparable to baseline values (0.04), indicating no significant antigen-specific response within these subclasses.

### 3.2. Chronic schistosomiasis does not alter the frequency of innate immune cells

To explore the effects of chronic schistosomiasis infection on innate immune cell responses to the HPV vaccine, we profiled cryopreserved PBMS samples obtained four weeks after the second HPV vaccine dose was administered. A flow cytometry assay was used. The innate immune cells were defined by their expressed cell surface markers. Monocyte subset populations were defined as: classical monocytes expressing CD3-CD14+ CD16-; intermediate monocytes (IM) expressing CD3-CD14+ CD16+; non-classical monocytes (NCM) expressing CD3-CD14-CD16+; and natural killer cells (NK cells) expressing CD3-CD16+ CD56+.

There were no significant differences in levels of classical, intermediate, and non-classical monocytes in all three animal groups after the two HPV vaccine doses were administered. Classical monocytes had generally lower levels in both the Schisto + HPV vaccine and Schisto + PZQ + HPV vaccine animal groups. However, the level of classical monocytes for two out of three animals in the HPV vaccine-only group was over 80%. In contrast, intermediate monocytes had lower levels in the HPV vaccine-only group, as compared to the Schisto + HPV vaccine and Schisto + PZQ + HPV vaccine animal groups, in which two out of three and three out of four animals had over 50% of intermediate monocytes (65% and 87% for Schisto + HPV vaccine and 57% and 59% for Schisto + PZQ + HPV vaccine), respectively.

**Figure 3.**
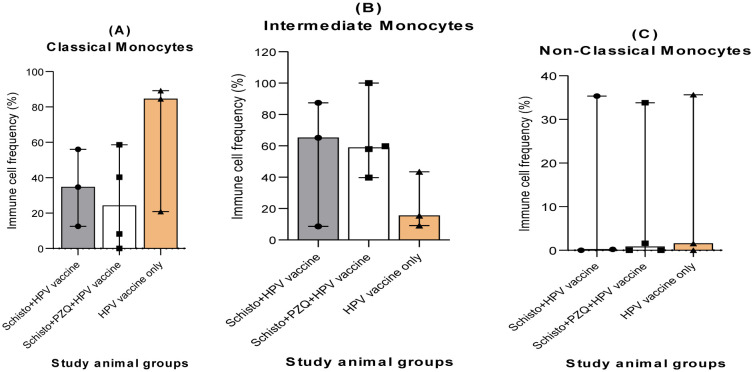
Frequency of circulating innate immune cells four weeks after the second HPV vaccine administration in Olive baboons for the three study groups: Schisto + HPV vaccine, Schisto + PZQ + HPV, HPV vaccine-only. Monocyte subset populations, classical monocytes A), intermediate monocytes (B), and non-classical monocytes (C) were expressed as a percentage of total monocyte populations. Descriptive statistics were used in innate immune cell analysis. Frequencies of monocytes were obtained from histogram analysis of PBMC populations on FlowJo version 10.10.0. Statistical analysis of the immune cell responses was done using Kruskal-Wallis. Selection criteria for statistical analysis is provided in supplementary Table S1. Descriptive statistics and individual animal data on innate immune cells are available in Table S2 and Table S4, respectively. Statistical significance was set at P < 0.05.

The distribution of non-classical monocytes and NK cells varied across the three experimental groups. In Group 1 (n = 3), one animal exhibited 4% non-classical monocytes while the remaining two animals had undetectable levels (0%). Additionally, a different animal within this group showed 6% NK cells, whereas the other two animals had 0% NK cell counts. In Group 2 (n = 4), one animal had a notably higher frequency of non-classical monocytes at 13%, with the remaining three animals showing 0%. All animals in Group 2 had undetectable NK cells (0%). In Group 3 (n = 3), none of the animals exhibited detectable levels of either non-classical monocytes or NK cells (Figure 3).

### 3.3. Chronic schistosomiasis infection does not alter HPV-specific adaptive immune cell levels

**Figure 4.**
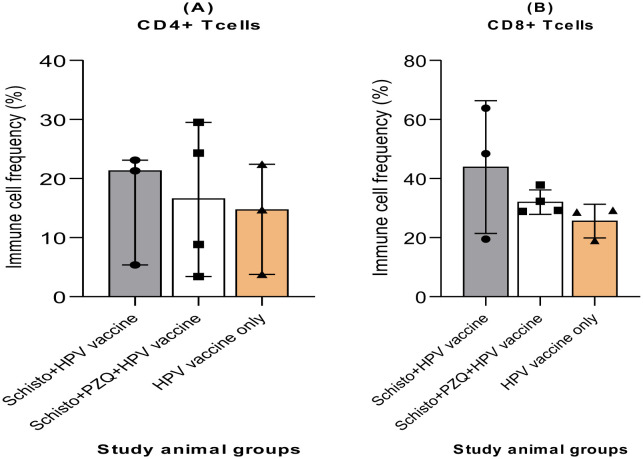
Frequency of circulating adaptive immune cells four weeks after second HPV vaccine administration in Olive baboons for the three study groups: Schisto + HPV vaccine, Schisto + PZQ+ HPV vaccine, HPV vaccine-only. Descriptive statistics were used in adaptive immune cell analysis. Frequencies of CD4+ and CD8+ T cells were obtained from histogram analysis of PBMC populations on Flowjo version 10.10.0. Statistical analysis to identify between-group differences in immune cell responses was done using Kruskall-Wallis. Selection criteria for statistical analysis is provided in supplementary Table S1. Descriptive statistics and individual animal data on innate immune cells are available in Table S2 and Table S4, respectively. Statistical significance was set at P < 0.05.

To explore the effects of chronic schistosomiasis infection on adaptive immune cell responses to the HPV vaccine, we profiled cryopreserved PBMS samples obtained four weeks after the second HPV vaccine dose was administered. A flow cytometry assay was used. T cell populations were defined as: CD4+ T cells expressing CD3+CD4+; and CD8+ T cells expressing CD3+CD8+. There were no statistically significant differences in T-cell population frequencies among the study groups (Figure 4). Adaptive immune responses exhibited varied frequencies across all three animal groups. CD4+ T cells had generally low-frequency levels across all animal groups: Schisto + HPV vaccine (16%), Schisto + PZQ + HPV vaccine (16%), and the HPV vaccine -only (13%). CD8+ equally exhibited varied cell frequencies: Schisto + HPV vaccine (43%), Schisto + PZQ + HPV vaccine (35%), and HPV vaccine -only (25%). Our observations indicate that chronic schistosomiasis infection does not affect HPV vaccine-induced adaptive immune cell frequencies.

### 3.4. Chronic Schistosomiasis has no significant effect on HPV-stimulated IL-1β and TNF-α cytokine concentrations

**Figure 5.**
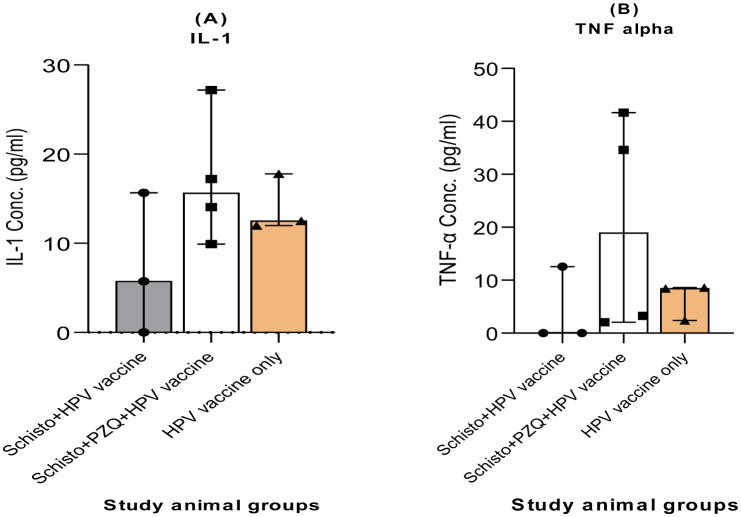
Production of IL-1β and TNF-α cytokine concentrations in 72-hour cell culture supernatants during specific stimulation with HPV Ag. PBMC cell culture supernatants were analyzed using cytokine ELISA. The bars represent the net IL-1β (A) and TNF-α (B) concentrations for each animal group. Statistical analysis to identify between group differences in cytokine concentration was done using Kruskall-Wallis test. Selection criteria for statistical analysis is provided in supplementary Table S1. Descriptive statistics and individual animal data on innate immune cells are available in Table S2 and Table S5, respectively. Differences were set to be statistically significant at P < 0.05.

The 72-hour cell culture supernatants were analyzed to determine the effect of chronic *Schistosomiasis* infection IL-1β and TNF-α cytokine concentrations (Figure 5). Cytokine ELISA was performed on cell culture supernatants from the three animal study groups; Schisto + HPV vaccine, Schisto + PZQ + HPV vaccine, and HPV vaccine-only. Cytokine concentrations were higher in the Schisto + PZQ + HPV vaccine for IL-1β (17.09 pg/mL) compared to Schisto + HPV vaccine (7.16 pg/mL) and HPV vaccine-only (14.1 pg/mL). Similarly, cytokine concentrations were higher in the Schisto + PZQ + HPV vaccine for TNF-α (20.39 pg/mL) compared to Schisto + HPV vaccine (4.26 pg/mL) and HPV vaccine-only (6.48 pg/mL). There were no statistically significant differences in IL-1β and TNF-α cytokine concentrations among the study groups. This statistic implies that chronic *Schistosomiasis* infection does not affect HPV vaccine-induced IL-1β and TNF-α cytokine concentrations.

## 4. Discussion

Prophylactic vaccines against human papillomaviruses are formulated using virus-like particles derived from the L1 protein, which induce HPV strain-specific antibodies and cellular immune responses [27]. This study investigated the impact of chronic schistosomiasis infection on HPV vaccine immune responses. The study demonstrated both innate and adaptive immune responses in baboons chronically infected with schistosomiasis, then vaccinated with the HPV vaccine; baboons chronically infected with schistosomiasis and treated with Praziquantel before the HPV vaccine administration; and baboons with the HPV vaccine only. The innate and adaptive immune responses included: HPV-specific whole IgG and IgG subclasses responses; monocyte subclasses and natural killer cells responses; CD4+ and CD8+ T cell responses; and TNF-α and IL-1 cytokine responses.

After two HPV vaccine doses were administered 4 weeks apart, we observed a generally significant increase in HPV-specific whole IgG and IgG1 in both the Schisto + HPV vaccine, Schisto + PZQ + HPV vaccine, and HPV vaccine-only groups. This significant increase in HPV-specific whole IgG levels was observed in the Schisto + PZQ + HPV vaccine group, 4 weeks after the first HPV vaccine dose was administered and four weeks after the second HPV vaccine dose was administered. This indicates that clearance of schistosomiasis infection before administering the HPV vaccine aids in mounting a humoral response against HPV infections, and this response can be enhanced with a booster dose. Clearance of schistosomiasis infection with Praziquantel before vaccination was also observed to restore whole IgG responses specific to HPV16 and HPV18 in a baboon model [11]. Similarly, treatment of schistosomiasis with Praziquantel before vaccination was reported to mildly restore HPV16-specific IgG responses in human subjects [15]. The Schisto + HPV vaccine and the HPV-only group exhibited a significant increase in HPV-specific whole IgG levels only after the second HPV vaccine dose was administered. This delay in the humoral response indicates that a booster dose of the HPV vaccine is essential for protection against infections with HPV types 16 and 18 for persons with helminthic infection.

We also observed a delayed significance in HPV-specific IgG1 antibody response in all animal groups after the first HPV vaccine dose, and it was only significant after the second HPV vaccine dose was administered. Several single-dose HPV vaccine efficacy study findings, including one by Baisley and colleagues [13], support the efficacy of a single-dose HPV vaccine. They observed that the HPV vaccine elicited comparable HPV16 and HPV18 IgG antibody responses in young girls and young women, one year after one dose of the bivalent or nanovalent HPV vaccine, suggesting that a single dose of the vaccines may protect against persistent HPV16 and HPV18 infection for a minimum of two years. This could improve vaccination rates in resource-limited settings. In this non-human primate study, we observed that whole IgG and IgG1 responses require a booster dose for optimal levels in animals chronically infected with schistosomiasis. Our findings do not contradict these studies but highlight the potential of helminth-induced immune modulation in impacting single-dose HPV-vaccine-induced immunity. Our observations suggest that booster doses might enhance protective responses in helminth-endemic populations.

Overall, our observations indicate that study subjects with chronic schistosomiasis infections can elicit HPV-specific whole IgG and IgG1 antibodies in response to the vaccines. The observed response of increased HPV-specific antibody levels could provide neutralization and opsonization defenses, preventing the spread of infection with HPV types 16 and 18. The differences in immune responses in comparison to previous findings could also be attributed to the different vaccine antigens, as the Cervarix vaccine was previously used to vaccinate the animals [11], while the Gardasil vaccine was used for the immune recall response for this current study.

Monocytes are key components of innate immunity and serve as the first line of defense against infection [28]; classical monocytes aid in phagocytosis, intermediate monocytes function in antigen presentation of antigens to immune cells, while non-classical monocytes help with complement-mediated phagocytosis of antigens [29],[30]. We observed generally lower levels of classical monocytes, intermediate monocytes, and non-classical monocytes than total monocytes. Classical monocytes had slightly higher expression in the HPV-only group than in the Schisto + HPV vaccine and the Schisto + PZQ + HPV vaccine groups. The level of classical monocytes for two out of three animals in the HPV-only group was over 80%, which is within the reported threshold of monocytes at 80–90% of total monocytes in humans [31]–[33]. Intermediate monocytes had slightly higher expression in the Schisto+HPV vaccine and the Schisto + PZQ + HPV vaccine groups, in which 2/3 and 3/4 animals had over 50% of intermedi#ate monocytes. These levels are however elevated when compared to the threshold of human intermediate monocytes as 5–10% of total monocytes [31]–[33]. Our observations implied that treated or chronic schistosomiasis did not alter the frequency of circulating monocyte populations in baboons after a two-dose HPV vaccine. In this study, the lack of significant immune cell responses could be attributed to the small sample size per animal group and outlier data in the immune cell frequencies that could have skewed the outcome. Overall, this study exhibited a low frequency of peripheral monocytes post-HPV vaccination in all three groups. This is contrary to a HPV vaccine study on human subjects that observed a generally higher count of circulating monocytes after bivalent vaccination of premenopausal HPV-seronegative women [30]. In another human study on HPV vaccine-induced immune responses, the bivalent HPV vaccine cohort showed a significant increase in intermediate monocytes, 1 day post first vaccine dose, while classical monocytes increased significantly, one day post third vaccine dose [34]. In both studies, subjects were not chronically infected with schistosomiasis.

The distribution of non-classical monocytes and NK cells varied across the three experimental groups. In Group 1 (n = 3), one animal exhibited 4% non-classical monocytes while the remaining two animals had undetectable levels (0%). Additionally, a different animal within this group showed 6% NK cells, whereas the other two animals had 0% NK cell counts. In Group 2 (n = 4), one animal had a notably higher frequency of non-classical monocytes at 13%, with the remaining three animals showing 0%. All animals in Group 2 had undetectable NK cells (0%). In Group 3 (n = 3), none of the animals exhibited detectable levels of either non-classical monocytes or NK cells. These findings suggest considerable inter-individual variability in the detection of non-classical monocytes and NK cells, with isolated elevations observed in Groups 1 and 2. The absence of these cell populations in Group 3 and in the majority of animals across all groups may reflect low basal expression levels in this model, technical limitations in detection or biological variability. Further investigation is required to determine whether these differences are biologically significant or stochastic.

CD4+ T cells aid adaptive immunity by recognizing antigens through T cell receptors, protecting against pathogens, and recruiting effector immune cells [35]. CD8+ T cells control viral infections by directly killing infected cells [36]. In this study, we observed low frequencies of both CD4+ and CD8+ T cells in all animal groups. Even though CD8+ T cell frequencies were higher than CD4+ T cells, both were not statistically significant. This was similarly exhibited in a SARS-CoV-2 vaccine efficacy study where mice infected with *Heligmosomoides polygyrus bakeri* (Hpb) showed reduced CD4+ and CD8+ T cell responses to an mRNA vaccine against the Wuhan-1 spike protein of SARS-CoV-2 (Desai et al., 2024). Our findings indicated that treated or chronic schistosomiasis did not alter the frequency of circulating CD4+ and CD8+ T cell populations in baboons after a two-dose HPV vaccine. The lack of significant immune cell responses could be attributed to the small study sample size (n = 3; n = 4) and outlier data in the immune cell frequencies that could have skewed the outcome.

This study also aimed to determine the effect of chronic *schistosomiasis* on HPV-induced cytokine responses. We observed slightly high cytokine concentrations of both IL-1β and TNF-α in the Schisto + PZQ + HPV vaccine compared to the Schisto + HPV vaccine and HPV vaccine-only groups. These differences were not significant. We found that treated or chronic schistosomiasis did not alter the production of IL-1β and TNF-α in baboons after a two-dose HPV vaccine. In a human cohort study on HPV16 L1 VLP-induced immune responses a similar observation was made where there were no significant increase IL-1β concentrations in PBMC culture supernatants. Other human studies observed a significant increase in TNF-α concentrations. A study on HPV16 L1 induced-cytokine responses in a phase II clinical trial on Novovax vaccine, observed an increase in TNF-α eight weeks post vaccination [37]. A study by Pinto et al., observed and increase in TNF-α concentrations in PBMC cell culture supernatants stimulated with HPV16 L1 VLPS, in comparison to the placebo group [38]. Goncalves et al observed an increase in TNF-a messenger RNA (mRNA) transcripts from human cohort PBMC samples, collected one month after three doses of bivalent HPV16/18 vaccination [39]. However, in all three human studies, there were no underlying helminth infections at the time of vaccination.

The study animals were initially vaccinated with two doses of Cervarix® which targets only HPV types 16 and 18, administered one month apart. For this current study, the Gardasil® vaccine consisting of 4 HPV antigens (HPV-16/18/6/11) was used, and for ELISA and cell culture assays focuses on the HPV16 and HPV18 antigens. Both vaccines protect against HPV16 and HPV18. However, both vaccines undergo different production systems; for Cervarix®, the L1 VLP protein is produced in insect cell lines, while for Gardasil®, the L1 VLP protein is produced from yeast. The different systems of production utilized for the 2 vaccines may cause differences in particle composition and epitope presentation. Also, the two vaccines exhibit differences in antigen composition. The differences in antigen composition may contribute to immunological interference and should be acknowledged as a limitation.

There is however no indication of Schistosomiasis resulting in reduction of HPV vaccine induced cellular responses in this study. While baboons exhibit similarities with humans, inherent differences may impact immune responses. Also, future studies with a larger sample size would help establish definitive interactions between *S. mansoni* and HPV vaccine efficacy.

## 5. Conclusions

This study highlights the interplay between helminth infections, vaccination, and immune responses. The observed increase in HPV-specific whole IgG antibody levels in animals treated for schistosomiasis before HPV vaccination underscores the importance of addressing helminth infections before vaccination. Additionally, the delayed HPV-specific IgG1 antibody response after the first vaccine dose, with significant levels only achieved after the booster dose, emphasizes the critical role of a booster in eliciting a strong antibody response. These findings suggest the potential for successful HPV vaccination in parasitic endemic regions. Implementing helminth treatment policies before HPV vaccination may enhance vaccine-induced responses.

## Use of AI tools declaration

No A.I tools in this manuscript.


