# Supplementary material for: Human Papillomavirus Vaccine immune responses in an Olive baboon model is not compromised by chronic Schistosoma mansoni infections
Source: AIMS Microbiol. 2025 Aug 7;11(3):720–36. doi: 10.3934/microbiol.2025030 (PMC12511963; doi:10.3934/microbiol.2025030)
Supplement: Supplementary file 1 [file microbiol-11-03-030-s001.pdf]

AIMS Microbiology, 11(3): 720–736.
DOI: 10.3934/microbiol.2025030
Received: 09 March 2025
Revised: 02 July 2025
Accepted: 15 July 2025
Published: 07 August 2025

https://www.aimspress.com/journal/microbiology

Research article

Human Papillomavirus Vaccine immune responses in an Olive baboon model is not compromised by chronic *Schistosoma mansoni* infections

Linda Obiero[1,2,]*, Edinah Songoro[1], Martin Omondi[2], Ruth Nyakundi[2,]* and Lucy Ochola[2,]*

[1] Jomo Kenyatta University of Agriculture and Technology
[2] Kenya Institute of Primate Research

* **Correspondence:** Email: obiero.linda@students.jkuat.ac.ke, nyakundirk@gmail.com, laochola@gmail.com.

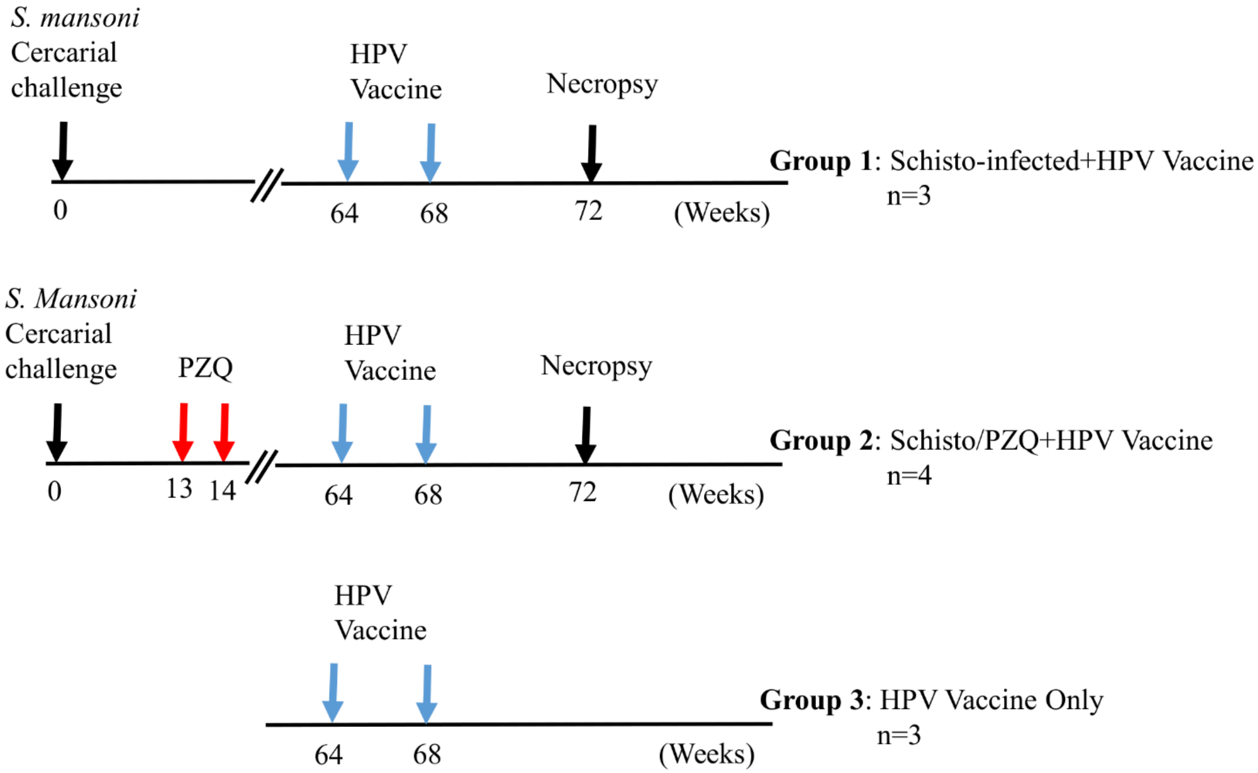

Figure S1. This is an experimental design for the previous study [11]: Three groups of animals were included in the experiment. i) animals infected with S. mansoni cercariae and later received the *Cervarix* HPV vaccine (Schisto infected+HPV), ii) animals infected with S. mansoni, treated with praziquantel (PZQ) before receiving the HPV vaccine (Schisto/PZQ+HPV) and iii) animals that received the HPV vaccine only.

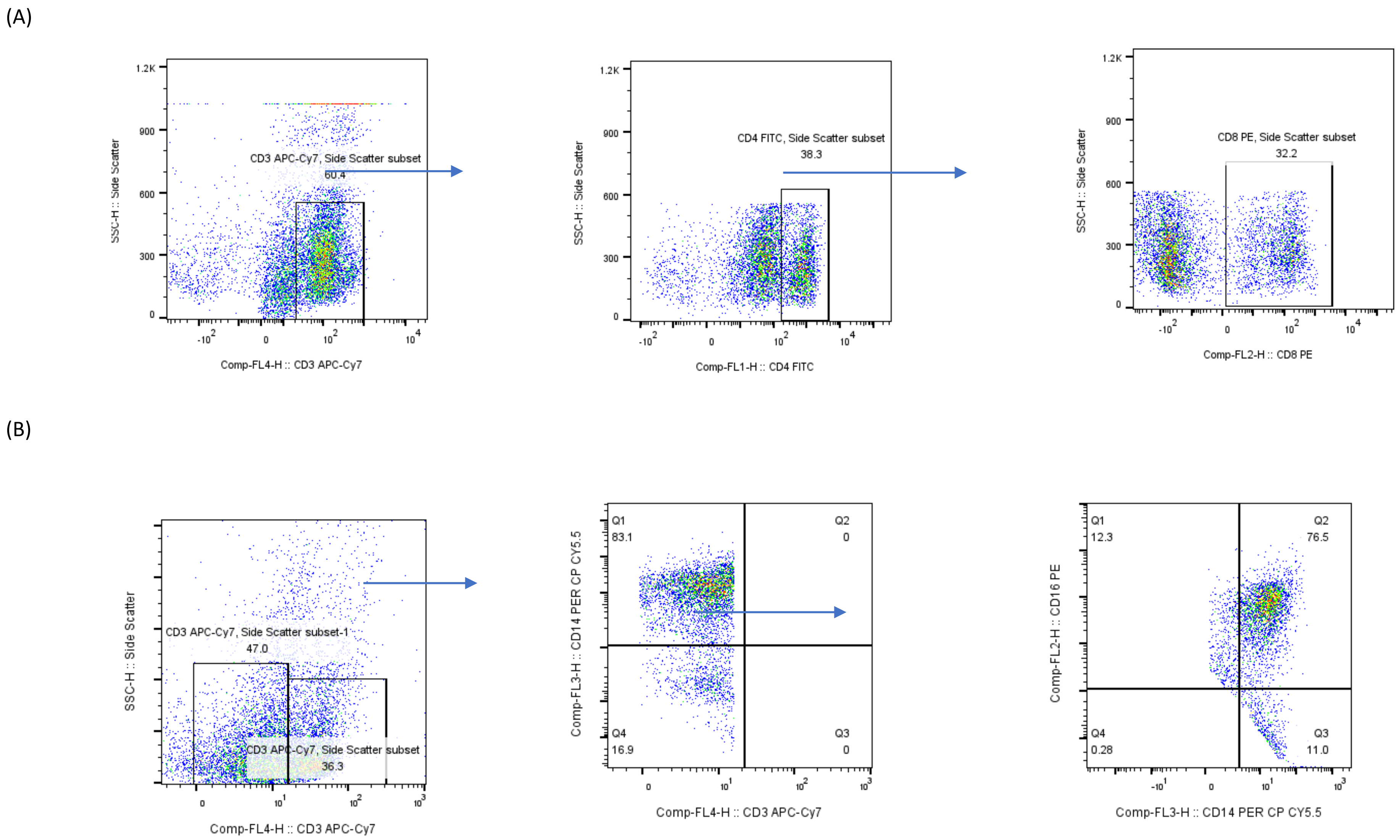
(A)
1.2K
900
600
300
0
SSC-H :: Side Scatter
CD3 APC-Cy7, Side Scatter subset
60.4
-10^2
0
10^2
10^4
Comp-FL4-H :: CD3 APC-Cy7
CD4 FITC, Side Scatter subset
38.3
Comp-FL1-H :: CD4 FITC
CD8 PE, Side Scatter subset
32.2
Comp-FL2-H :: CD8 PE
(B)
CD3 APC-Cy7, Side Scatter subset-1
47.0
CD3 APC-Cy7, Side Scatter subset
36.3
10^1
10^2
10^3
Q1
83.1
Q2
0
Q4
16.9
Q3
0
Comp-FL3-H :: CD14 PER CP CY5.5
Q1
12.3
Q2
76.5
Q4
0.28
Q3
11.0
-10^1
Comp-FL2-H :: CD16 PE

(C)

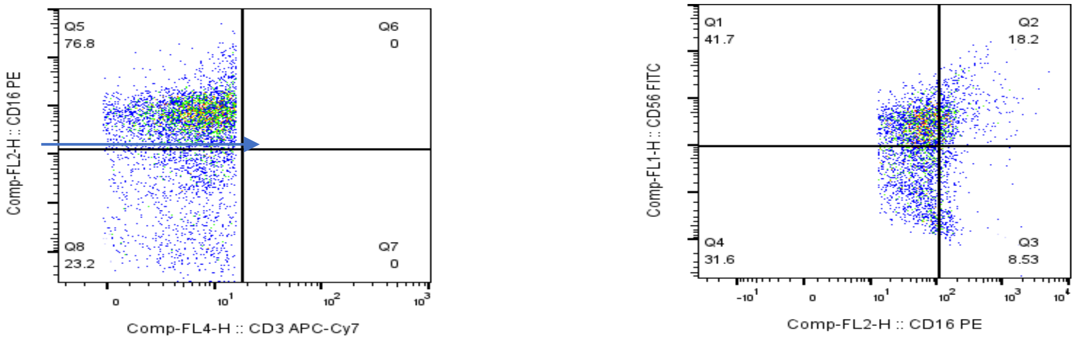

Figure S2. Gating strategy for innate and adaptive immune cells: T cells were analyzed as CD3+ CD4+ and CD3+CD8+, from total CD3+ cells (A). Monocytes were analyzed as classical monocytes expressing CD3-CD14+ CD16-; intermediate monocytes (IM) expressing CD3-CD14+ CD16+; non-classical monocytes (NCM) expressing CD3-CD14-CD16+; from total CD3- cells (B). NK cells were analyzed as CD3-CD16+CD56+ from total CD3- cells (C).

Table S1. Selection criteria for analysis: All statistical analyses were done using GraphPad Prism version 8.0.2.263 (GraphPad Software, Inc.). Differences in immune responses were measured between and within all groups; at baseline, before the HPV vaccine dose (T0), four weeks after the first HPV vaccine dose (T1), and four weeks after the second vaccine dose (T2). Differences in antibody levels (IgG and IgG1) were measured between and within all groups; at baseline (T0), four weeks after the first HPV vaccine dose (T1), and four weeks after the second vaccine dose (T2). Differences in the expression of immune cells (monocytes, NK cells, CD4+ T cells, and CD8+ T cells) were measured between groups only, at T2 (four weeks after the second HPV vaccine dose). The Shapiro-Wilk Test was used to determine whether data was normally or non-normally distributed. Both normally and non-normally distributed data were analyzed by the Kruskal-Wallis test with Dunn's post hoc test due to the small group-n number (n=3; n=4 for animal groups and n=10 for all groups). The p-value for statistical significance was set at P<0.05.

Analysis Conducted	Test variables	N	Normality test (Shapiro Wilk test)	Statistical test selected (Friedman test)	Statistical test selected (Kruskall-Wallis test with Dunn's post hoc)
IgG	Between groups - Schisto+HPV vs. Schisto+PZQ+HPV			>0.9999	
	Between groups - Schisto+HPV vs. HPV only			>0.9999	
	Between groups - Schisto+PZQ+HPV vs. HPV only			>0.9999	
	Within group-Schisto+HPV (T0, T1, T2)	3	0.0006		
	• T0 VS. T1				0.1363
	• T0 VS. T2				0.0004
	• T1 VS. T2				0.1980
	Within group-Schisto+PZQ+HPV (T0, T1, T2)	4	0.74		
	• T0 VS. T1				0.0100
	• T0 VS. T2				0.0003
	• T1 VS. T2				>0.9999
	Within group-HPV only (T0, T1, T2)	3	0.0004		0.0242
	• T0 VS. T1				0.0331
	• T0 VS. T2				>0.9999
	• T1 VS. T2				

Continued on nexr page

Analysis Conducted	Test variables	N	Normality test (Shapiro Wilk test)	Statistical test selected (Friedman test)	Statistical test selected (Kruskall-Wallis test with Dunn's post hoc)
IgG1	Between groups - Schisto+HPV vs. Schisto+PZQ+HPV			0.6620	
				0.6620	
	Between groups - Schisto+HPV vs. HPV only			>0.9999	
	Between groups - Schisto+PZQ+HPV vs. HPV only	3	0.0687		
	Within group-Schisto+HPV (T0, T1, T2)				0.0799
	• T0 VS. T1				0.0016
	• T0 VS. T2				0.6408
	• T1 VS. T2	4			
			0.0043		
	Within group-Schisto+PZQ+HPV (T0, T1, T2)				0.0708
	• T0 VS. T1				<0.0001
	• T0 VS. T2				0.0708
	• T1 VS. T2				
		3			
	Within group-HPV only (T0, T1, T2)				0.9912
			0.0175		
	• T0 VS. T1				0.0105
	• T0 VS. T2				0.1547
	• T1 VS. T2				

Continued on nexr page

Analysis Conducted	Test variables	N	Normality test (Shapiro Wilk test)	Statistical test selected (Friedman test)	Statistical test selected (Kruskall-Wallis test with Dunn's post hoc)
Total monocytes	Between groups - Schisto+HPV vs. Schisto+PZQ+HPV	10	0.5092		>0.9999
					>0.9999
	Between groups - Schisto+HPV vs. HPV only				0.6614
	Between groups - Schisto+PZQ+HPV vs. HPV only				
Classical monocytes	Between groups - Schisto+HPV vs. Schisto+PZQ+HPV	10	0.5390		>0.9999
					0.8421
	Between groups - Schisto+HPV vs. HPV only				0.4186
	Between groups - Schisto+PZQ+HPV vs. HPV only				
Intermediate monocytes	Between groups - Schisto+HPV vs. Schisto+PZQ+HPV	10	0.5343		>0.9999
					0.8421
	Between groups - Schisto+HPV vs. HPV only				0.4186
	Between groups - Schisto+PZQ+HPV vs. HPV only				
Non-classical monocytes	Between groups - Schisto+HPV vs. Schisto+PZQ+HPV	10	0.0001		>0.9999
					>0.9999
	Between groups - Schisto+HPV vs. HPV only				>0.9999
	Between groups - Schisto+PZQ+HPV vs. HPV only				

Continued on nexr page

Analysis Conducted	Test variables	N	Normality test (Shapiro Wilk test)	Statistical test selected (Friedman test)	Statistical test selected (Kruskall-Wallis test with Dunn's post hoc)
Natural killer cells	Between groups - Schisto+HPV vs. Schisto+PZQ+HPV	10	<0.0001		>0.999
	Between groups - Schisto+HPV vs. HPV only				0.838
	Between groups - Schisto+PZQ+HPV vs. HPV only				0.129
CD4+ T cells	Between groups - Schisto+HPV vs. Schisto+PZQ+HPV	10	0.1866		>0.999
	Between groups - Schisto+HPV vs. HPV only				>0.999
	Between groups - Schisto+PZQ+HPV vs. HPV only				>0.999
CD8+ T cells	Between groups - Schisto+HPV vs. Schisto+PZQ+HPV	10	0.0718		>0.999
	Between groups - Schisto+HPV vs. HPV only				0.360
	Between groups - Schisto+PZQ+HPV vs. HPV only				0.598
IL-1β	Between groups - Schisto+HPV vs. Schisto+PZQ+HPV	10	0.901		0.419
	Between groups - Schisto+HPV vs. HPV only				0.842
	Between groups - Schisto+PZQ+HPV vs. HPV only				>.999

Continued on nexr page

Analysis Conducted	Test variables	N	Normality test (Shapiro Wilk test)	Statistical test selected (Friedman test)	Statistical test selected (Kruskall-Wallis test with Dunn’s post hoc)
	Between groups - Schisto+HPV vs. Schisto+PZQ+HPV	10	0.004		0.543
					>0.999
	Between groups - Schisto+HPV vs. HPV only				>0.999
	Between groups - Schisto+PZQ+HPV vs. HPV only				

Table S2. Descriptive statistics for all presented results. All descriptive statistics were analyzed using Graph Pad Prism software Version 8.00 for Windows.

1. IgG

Schisto + HPV vaccine			
Kruskal-Wallis Test<br>Descriptive statistics	T0	T1	T2
Number of values	6	6	6
Minimum	0.5710	1.916	2.248
25% Percentile	0.6753	2.032	2.297
Median	0.7720	2.105	2.356
75% Percentile	1.089	2.214	2.448
Maximum	1.910	2.251	2.510
Mean	0.9250	2.108	2.368
Std. Deviation	0.4906	0.1180	0.09317
Std. Error of Mean	0.2003	0.04819	0.03804
Lower 95% CI	0.4102	1.984	2.271
Upper 95% CI	1.440	2.232	2.466
Mean ranks	3.500	9.667	15.33

Schisto+PZQ+HPV vaccine			
Kruskal-Wallis Test<br>Descriptive statistics	To	T1	T2
Number of values	8	8	8
Minimum	0.5730	1.922	2.197
25% Percentile	0.6310	2.008	2.301
Median	0.7570	2.248	2.375
75% Percentile	0.9460	2.493	2.487
Maximum	1.067	2.642	2.508
Mean	0.7928	2.242	2.378
Std. Deviation	0.1744	0.2586	0.1099
Std. Error of Mean	0.06166	0.09143	0.03887
Lower 95% CI	0.6469	2.026	2.286
Upper 95% CI	0.9386	2.459	2.470
Mean ranks	4.500	14.88	18.13

HPV Vaccine only			
Kruskal-Wallis Test<br>Descriptive statistics	T0	T1	T2
Number of values	6	6	6
Minimum	0.7190	0.7750	2.386
25% Percentile	0.7595	1.264	2.391
Median	0.8475	2.484	2.431
75% Percentile	0.9793	2.548	2.486
Maximum	0.9800	2.549	2.502
Mean	0.8577	2.045	2.437
Std. Deviation	0.1061	0.7600	0.04722
Std. Error of Mean	0.04333	0.3103	0.01928
Lower 95% CI	0.7463	1.247	2.387
Upper 95% CI	0.9691	2.842	2.486
Mean ranks	4.167	12.33	12.00

2. IgG 1

Schisto + HPV vaccine			
Kruskal-Wallis Test<br>Descriptive statistics	T0	T1	T2
Number of values	6	6	6
Minimum	0.013	0.13	0.25
25% Percentile	0.024	0.17	0.28
Median	0.033	0.21	0.32
75% Percentile	0.069	0.31	0.35
Maximum	0.14	0.36	0.37
Mean	0.049	0.23	0.31
Std. Deviation	0.045	0.082	0.041
Std. Error of Mean	0.018	0.034	0.017
Lower 95% CI	0.0011	0.15	0.27
Upper 95% CI	0.096	0.32	0.36
Mean ranks	3.7	11	14

Schisto + PZQ + HPV vaccine			
Kruskal-Wallis Test<br>Descriptive statistics	T0	T1	T2
Number of values	8	8	8
Minimum	0.02900	0.2290	0.3950
25% Percentile	0.02925	0.2855	0.4085
Median	0.03150	0.3130	0.4395
75% Percentile	0.04750	0.3610	0.4933
Maximum	0.07300	0.3660	0.5290
Mean	0.03975	0.3139	0.4505
Std. Deviation	0.01549	0.04720	0.04786
Std. Error of Mean	0.005476	0.01669	0.01692
Lower 95% CI	0.02680	0.2744	0.4105
Upper 95% CI	0.05270	0.3533	0.4905
Mean ranks	4.500	12.50	20.50

HPV vaccine only			
Kruskal-Wallis Test<br>Descriptive statistics	T0	T1	T2
Number of values	6	6	6
Minimum	0.02500	0.01600	0.3400
25% Percentile	0.02575	0.02050	0.3543
Median	0.03700	0.2715	0.4260
75% Percentile	0.1203	0.3913	0.4798
Maximum	0.1330	0.4490	0.5210
Mean	0.06233	0.2337	0.4230
Std. Deviation	0.04893	0.1793	0.06704
Std. Error of Mean	0.01997	0.07322	0.02737
Lower 95% CI	0.01099	0.04546	0.3526
Upper 95% CI	0.1137	0.4219	0.4934
Mean ranks	5.500	8.500	14.50

3. Classical monocytes

Kruskal-Wallis Test<br>Descriptive statistics	Schisto+HPV vaccine	Schisto+PZQ+HPV vaccine	HPV vaccine only
Number of values	3	4	3
Minimum	12.55	0.000	20.87
25% Percentile	12.55	2.053	20.87
Median	34.67	24.23	84.56
75% Percentile	56.01	54.05	89.25
Maximum	56.01	58.64	89.25
Mean	34.41	26.78	64.89
Std. Deviation	21.73	27.44	38.20
Std. Error of Mean	12.55	13.72	22.05
Lower 95% CI	-19.58	-16.88	-29.99
Upper 95% CI	88.40	70.44	159.8
Mean ranks	5.000	4.250	7.667

4. Intermediate monocytes

Kruskal-Wallis Test<br>Descriptive statistics	Schisto+HPV vaccine	Schisto+PZQ+HPV vaccine	HPV vaccine only
Number of values	3	4	3
Minimum	8.635	39.74	9.186
25% Percentile	8.635	44.30	9.186
Median	65.11	58.86	15.44
75% Percentile	87.45	89.94	43.48
Maximum	87.45	100.0	43.48
Mean	53.73	64.36	22.70
Std. Deviation	40.62	25.42	18.26
Std. Error of Mean	23.45	12.71	10.54
Lower 95% CI	-47.18	23.91	-22.66
Upper 95% CI	154.6	104.8	68.07
Mean ranks	6.000	6.750	3.333

5. Non-classical monocytes

Kruskal-Wallis Test Descriptive statistics	Schisto+HPV vaccine	Schisto+PZQ+HPV vaccine	HPV vaccine only
Number of values	3	4	3
Minimum	0.000	0.000	0.000
25% Percentile	0.000	0.000	0.000
Median	0.2222	0.8102	1.566
75% Percentile	35.35	25.77	35.65
Maximum	35.35	33.82	35.65
Mean	11.86	8.859	12.41
Std. Deviation	20.35	16.66	20.15
Std. Error of Mean	11.75	8.328	11.63
Lower 95% CI	-38.68	-17.64	-37.64
Upper 95% CI	62.40	35.36	62.45
Mean ranks	5.500	5.000	6.167

6. NK Cells

Kruskal-Wallis Test Descriptive statistics	Schisto+HPV vaccine	Schisto+PZQ+HPV vaccine	HPV vaccine only
Number of values	3	4	3
Minimum	0.02000	0.3600	0.000
25% Percentile	0.02000	0.3750	0.000
Median	0.04000	0.4450	0.02000
75% Percentile	6.560	0.5900	0.3000
Maximum	6.560	0.6300	0.3000
Mean	2.207	0.4700	0.1067
Std. Deviation	3.770	0.1158	0.1677
Std. Error of Mean	2.177	0.05788	0.09684
Lower 95% CI	-7.159	0.2858	-0.3100
Upper 95% CI	11.57	0.6542	0.5233
Mean ranks	5.500	7.500	2.833

7. CD 4+ T cells

Kruskal-Wallis test Descriptive Statistics	Schisto+HPV vaccine	Schisto+PZQ+HPV vaccine	HPV vaccine only
Number of values	3	4	3
Minimum	5.390	3.400	3.780
25% Percentile	5.390	4.760	3.780
Median	21.30	16.57	14.70
75% Percentile	23.10	28.20	22.40
Maximum	23.10	29.50	22.40
Mean	16.60	16.51	13.63
Std. Deviation	9.747	12.38	9.356
Std. Error of Mean	5.627	6.192	5.402
Lower 95% CI	-7.616	-3.196	-9.616
Upper 95% CI	40.81	36.22	36.87
Mean ranks	5.667	6.000	4.667

8. CD 8+ T cells

Kruskal-Wallis Test Descriptive statistics	Schisto+HPV vaccine	Schisto+PZQ+HPV vaccine	HPV vaccine only
Number of values	3	4	3
Minimum	19.50	28.90	19.00
25% Percentile	19.50	28.98	19.00
Median	48.40	30.75	28.60
75% Percentile	63.80	36.43	29.20
Maximum	63.80	37.80	29.20
Mean	43.90	32.05	25.60
Std. Deviation	22.49	4.130	5.724
Std. Error of Mean	12.98	2.065	3.305
Lower 95% CI	-11.97	25.48	11.38
Upper 95% CI	99.77	38.62	39.82
Mean ranks	7.000	6.125	3.167

Table S3. Individual animal data on antibody optical densities (O.D's) for all study animal groups. All optical densities were obtained using the ELISA reader (Biotek Elx808) at 450 nm.

**IgG OPTICAL DENSITIES (450NM)**		
**T0**	**T1**	**T2**
0.815	2.201	2.248
0.571	2.071	2.388
0.735	2.138	2.313
0.71	1.916	2.324
0.809	2.251	2.427
1.91	2.07	2.51
0.956	2.044	2.342
0.916	2.245	2.306
0.719	2.566	2.508
0.712	2.642	2.408
0.573	2.273	2.299
0.604	1.996	2.197
1.067	1.922	2.493
0.795	2.251	2.468
0.979	2.47	2.48
0.98	2.498	2.444
0.852	2.549	2.417
0.843	2.548	2.502
0.773	1.427	2.386
0.719	0.775	2.392
**IgG1 OPTICAL DENSITIES (450NM)**		
**T0**	**T1**	**T2**
0.046	0.229	0.336
0.013	0.2	0.312
0.028	0.187	0.288
0.034	0.364	0.253
0.138	0.289	0.373
0.032	0.132	0.322
0.073	0.366	0.498
0.03	0.352	0.405
0.03	0.324	0.529
0.033	0.229	0.425
0.046	0.302	0.419
0.048	0.284	0.479
0.029	0.364	0.395
0.029	0.29	0.454
0.03	0.372	0.359
0.025	0.26	0.466
0.026	0.449	0.423

IgG1 OPTICAL DENSITIES (450NM)		
**T0**	**T1**	**T2**
0.116	0.283	0.429
0.044	0.022	0.34
0.133	0.016	0.521

Table S4. Individual animal data on immune cell frequencies for all study animal groups. All immune cell frequencies were obtained from histogram analysis of PBMC populations on FlowJo version 10.10.0.

animal group	Natural Killer cells (% frequency)	NON-Classical monocytes (% frequency)	Intermediate monocytes (% frequency)	CLASSICAL MONOCYTES (% frequency)	CD4+ T Cells (% frequency)	CD8+ TCELLS (% frequency)	Tregs (% frequency)	Tmem (% frequency)
Schisto+HPV	0.04	0.02	5.86	3.12	23.1	19.5	1.24	0.73
Schisto+HPV	6.56	0	29.9	4.29	5.39	48.4	0.53	5.72
Schisto+HPV	0.02	4.79	1.17	7.59	21.3	63.8	0.61	28.3
Schisto+PZQ+HPV	0.36	13.3	22.8	3.23	24.3	29.2	0.66	9.44
Schisto+PZQ+HPV	0.63	0	9.32	6.28	3.4	28.9	1.13	16.1
Schisto+PZQ+HPV	0.47	0	1.74	0	8.84	32.3	1.12	0.93
Schisto+PZQ+HPV	0.42	0.42	10.3	15.2	29.5	37.8	0.78	0.85
HPV only	0.02	0.41	0.5	0.24	14.7	28.6	0.74	1.85
HPV only	0	0	1.03	5.64	3.78	19	2.43	4.08
HPV only	0.3	0.3	1.76	17.1	22.4	29.2	0.76	19.2

Table S5. Individual animal data on cytokine concentrations (in pg/mL) for all study animal groups.

Animal group	IL-1β Net concentration (pg/ml)	TNF alpha Net Concentration (pg/mL)
Schisto+HPV	15.65098	0.1
Schisto+HPV	0.1	0.1
Schisto+HPV	5.739376	12.56594
Schisto+PZQ+HPV	9.914725	2.059609
Schisto+PZQ+HPV	17.20322	3.261486
Schisto+PZQ+HPV	27.18656	34.59857
Schisto+PZQ+HPV	14.06129	41.62892
HPV only	11.97663	2.408269
HPV only	12.51919	8.443771
HPV only	17.79125	8.591722

AIMS Press

© 2025 the Author(s), licensee AIMS Press. This is an open access article distributed under the terms of the Creative Commons Attribution License (https://creativecommons.org/licenses/by/4.0)
